# Reproductive Health in Scleroderma, Vasculitis, and Sjögren Syndrome

**DOI:** 10.1097/RHU.0000000000002128

**Published:** 2024-09-27

**Authors:** Francesca Crisafulli, Maria Grazia Lazzaroni, Cecilia Nalli, Rossana Orabona, Franco Franceschini, Angela Tincani

**Affiliations:** From the ∗ Rheumatology and Clinical Immunology Unit–ERN ReCONNET, Department of Clinical and Experimental Sciences, ASST Spedali Civili and University of Brescia, Brescia, Italy; † Obstetric and Gynecology Unit, ASST Spedali Civili of Brescia, Brescia, Italy.

**Keywords:** pregnancy, systemic sclerosis, vasculitis, Sjögren

## Abstract

Women with systemic chronic inflammatory disease, such as those with scleroderma, systemic vasculitis, and Sjögren syndrome, need preconception evaluation by a multidisciplinary team. Counseling and pregnancy management should be tailored to patients' needs, considering specific disease features, organ involvement, treatment options, and risk factors to minimize risks of maternal-fetal complications during pregnancy.

Additionally, considerations regarding fertility, assisted reproductive techniques, and contraception also need to be addressed for these women.

In this narrative review, we integrate the current published literature with our expert opinion to address the issues faced by patients with the aforementioned inflammatory conditions.

Women with systemic chronic inflammatory diseases such as those with scleroderma (SSc), Sjögren syndrome (SS), and systemic vasculitis (SV) need careful evaluation before conception. A preconceptional multidisciplinary risk assessment is a critical step to ensure a good outcome for a future pregnancy and should account for several issues (Fig.).

**FIGURE:**
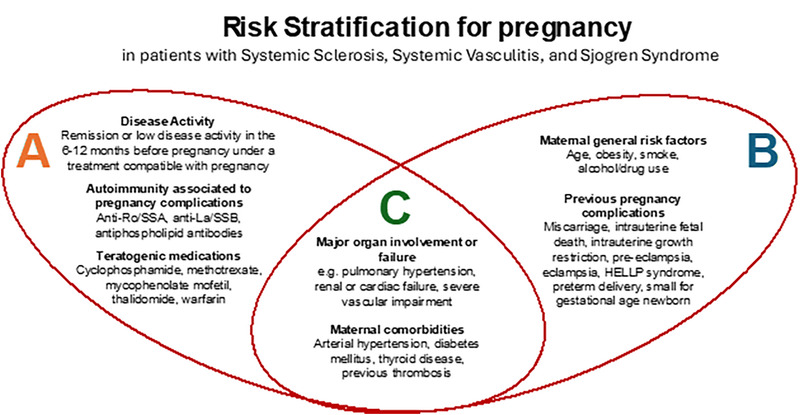
Risk stratification for pregnancy in patients with systemic sclerosis, systemic vasculitis, and Sjögren syndrome. A, Risk factors of rheumatological competence. B, Risk factors of obstetrical competence. C, Risk factors usually investigated by both specialists' groups. This picture underlines the importance of a multidisciplinary prepregnancy counseling.

The treating physician(s), including a rheumatologist and, if needed, other medical subspecialists, collaborate with the obstetric/gynecologist trained to manage high-risk pregnancies.

Disease-related risk factors, such as high titers anti-Ro/SS-A antibodies, current disease activity (which can be particularly high in patients with a recent diagnosis), and disease damage (such as pulmonary hypertension in SSc), which could negatively impact pregnancy, must be evaluated by the multidisciplinary team. In general, effective control of maternal disease activity prior to and during pregnancy is crucial, as well as the evaluation of both previous and current medication use. Indeed, previous use of certain medications might affect fertility, whereas some medications that used to control the disease might not be compatible with pregnancy and therefore require modification.^1^ Please refer to Siegel et al^1^ for additional information on medications in pregnancy and lactation in this supplement.

In addition to specific considerations related to the chronic inflammatory conditions discussed in this review, general risk factors should be assessed, as in the general obstetric population. These include age, smoking, alcohol consumption, prepregnancy body mass index, hypertension, diabetes, thyroid disease as well as other comorbidities, and obstetric history (Fig.).

## SYSTEMIC SCLEROSIS

SSc is a rare connective tissue disorder exhibiting high variability in clinical phenotype, severity, and prognosis. Cardiopulmonary involvement carries the highest burden of morbidity and mortality, with annual screening required in asymptomatic patients, even in low-risk subsets.^2^

In the last decades, pregnancy in women with SSc is becoming a more frequent event, leading to significant changes in counseling attitudes, improvements in disease knowledge, and increased availability of safe therapeutic options for both pregnancy and lactation.^3^

### Fertility

Although no rigorous case-control studies have been conducted to date, fertility rates in SSc patients, according to retrospective studies, seem similar to those of the general population.^4,5^

However, sexual dysfunction is a frequent issue in SSc and can negatively impact the reproductive plans of these women. In fact, fibrosis and vascular alteration of the genital tract can cause vaginal tightness and dryness, making sexual intercourse difficult and painful. Additionally, Raynaud phenomenon and digital ulcers, which are common manifestations of the disease, can also negatively affect sexual relationships.^3^

Assisted reproductive techniques (ARTs) are not contraindicated in SSc. Nevertheless, the individual thrombotic risk profile should be carefully assessed in each patient to recommend the optimal prophylaxis during estrogens exposure.^6^ Please refer to Leavitt et al^7^ for additional information on ART in this supplement.

Since the worldwide pooled prevalence of antiphospholipid antibodies (aPL) positivity is estimated at 14% (9–20%), a complete aPL profile should always be obtained.^8^

### Preconception Counseling

Optimal management of pregnancy in SSc should ideally start with preconception counseling in a multidisciplinary pregnancy clinic (Fig.). Every patient should undergo pulmonary function tests and echocardiography updated at the time this counseling occurs, as cardiopulmonary involvement requires special consideration and can also represent a contraindication to pregnancy. Currently, the main contraindications are represented by the following conditions:

- Pulmonary hypertension. This complication affects a limited subgroup of patients, more frequently in the late phase of the disease, but carries the highest risk of mortality and morbidity during pregnancy, for both the mother and the fetus. If an unplanned pregnancy occurs, the option of early termination should be considered. For women deciding to continue their pregnancy despite the risks, referral to expert centers should be recommended.^9^
- Severe restrictive lung disease. Considering the impact of the late phases of pregnancy on lung volume reduction, pregnancy avoidance should be advised for all patients with a significant baseline reduction in lung volumes, especially for forced vital capacity ≤50%.^10^
- Severe cardiac involvement. Moderate to severe impairment in cardiac function, leading to reduced left ventricular ejection fraction and functional limitation (New York Heart Association, stages 3 and 4), is considered a contraindication to pregnancy.^11^

Rarely, preconception counseling for a woman who has had a previous scleroderma renal crisis is needed. Some providers recommend a trial off angiotensin-converting enzyme inhibitors before pregnancy, with a possible switch to other antihypertensive drugs compatible with pregnancy, such as nifedipine.^12,13^ If blood pressure control is not achieved, and the patient is still determined to try to get pregnant, then angiotensin-converting enzyme inhibitors should be restarted and continued throughout gestation after a carefully considered shared decision-making process, including discussion of the risks and benefits of continuing this medication during pregnancy.^12^

Special attention should also be directed toward women with recent onset of SSc, especially those with “high-risk features” such as rapidly progressing diffuse cutaneous involvement (dcSSc) and anti-topoisomerase I positivity, since impending disease progression, including interstitial lung disease, may develop, and pregnancy and the postpartum period have also been suggested as possible accelerators of this process.^14^

### Pregnancy

According to the existing literature, a stable disease course during pregnancy has been described. Noteworthy, most studies have a retrospective design and include patients with heterogenous clinical phenotype, reflecting real-world experience. Therefore, it is possible that patients with severe organ involvement could receive counseling against pregnancy, thus creating a selection bias toward patients with a milder disease phenotype and a relative lack of information for those with a more severe phenotype.^3^

Improvement in vascular manifestations (eg, Raynaud phenomenon, digital ulcers) is often observed during pregnancy, whereas gastroesophageal symptoms worsen. Dyspnea and lower limbs edema, which can occur in the later stages even in healthy pregnancies, can further complicate the detection of worsening cardiopulmonary manifestations. Careful clinical examination should include the evaluation of oxygen saturation, and monitoring of laboratory parameters (N-terminal pro-b-type natriuretic peptide; uric acid) should be part of the routine follow-up. Additionally, echocardiography, electrocardiogram, and cardiac magnetic resonance imaging should be requested when additional information is needed.

Management is further complicated by the challenge of differential diagnosis between SSc manifestations and obstetric complications (eg, scleroderma renal crisis vs preeclampsia), where collaboration between specialists is critical and should also include the acquisition of data from maternal-fetal Doppler ultrasound.^15^

If immunosuppressive drugs are needed to control maternal disease during pregnancy, especially with the indication of lung or dcSSc, azathioprine is a safe option even though no formal data can support this approach. Rituximab can be used until a positive pregnancy test. In case of life- or organ-threatening disease, it may be used during pregnancy after a multidisciplinary evaluation and a shared decision-making process with the patient. Symptomatic treatment of Raynaud phenomenon and its complications can safely include calcium channel blockers, whereas intravenous iloprost should be stopped at the time of a positive pregnancy test.^6,13,16,17^

### Obstetric and Fetal Outcome

Over the last decades, these pregnancies have shown a decreasing trend in obstetric complications, although an increased risk compared with the healthy population is still recorded.

A meta-analysis carried out in 2019, which included 16 studies involving 1403 pregnancies in SSc patients, half of whom had dcSSc but with a low frequency of organ involvement, highlighted a higher risk of obstetric complications compared with the general obstetric population,^18^ as reported in Table 1:

**TABLE 1:** Results of the Meta-analysis of Obstetric Complications in SSc From Blagojevic et al^18^

Outcome	OR	95% CI	Comments
Miscarriages	1.6	1.22–2.22	No correction for disease severity or concomitant obstetric conditions
IUGR	3.2	2.21–4.53	
Preterm delivery <37 weeks	2.4	1.14–4.86	Corticosteroids identified as risk factors in 1 study^19^
Low birth weight	3.8	2.16–6.56

- miscarriages (odds ratio [OR], 1.6; 95% confidence interval [CI], 1.22–2.22), even if not acknowledging disease severity or concomitant obstetric conditions;
- intrauterine growth restriction (IUGR) (OR, 3.2; 95% CI, 2.21–4.53);
- preterm delivery (OR, 2.4; 95% CI, 1.14–4.86), including severe preterm delivery <34 weeks, with corticosteroids identified as risk factors in 1 study^19^; and
- low birth weight (OR, 3.8; 95% CI, 2.16–6.56).

The association with preeclampsia was highlighted in the majority of the studies, including 2 prospective cohorts. Importantly, a multicenter case-control study (published in abstract form) highlighted association with the following risk factors: baseline arterial hypertension, use of immunosuppressive agents or iloprost, twin pregnancy, and ARTs.^20^

Regarding delivery modality, a higher rate of cesarean section is observed in SSc patients, with a frequency ranging around 50%–65%, approximately double that observed in the general population.^3^ This highlights the importance of involving the obstetricians in the decision-making process, considering SSc-related factors (eg, musculoskeletal involvement, cardiopulmonary distress) and purely obstetric factors.

During the postpartum period, patients with SSc remain at higher risk of developing complications. Indeed, according to the results of a recent systematic literature review (SLR) and meta-analysis, 1 or more disease manifestations worsened or appeared in 10.5% if cases during the 6 months postpartum period. The worsening was mainly related to peripheral vascular and skin involvement, but in 2 cases, it resulted in maternal death (due to scleroderma renal crisis and aspiration pneumonia complicated by multiorgan failure).^18^ Therefore, clinical evaluation along with assessment of renal function, blood pressure, and cardiac function remains crucial also after delivery.^21^

## SYSTEMIC VASCULITIS

SV is a heterogenous group of rare and potentially life-threatening diseases characterized by inflammation of blood vessels, which can affect virtually any organ and may result in tissue damage and organ failure.^22^

Among the different forms of vasculitis, those most commonly diagnosed in women of childbearing age are Takayasu arteritis (TAK), Behçet disease (BD), and polyarteritis nodosa.^23,24^ ANCA-associated vasculitis (AAV), despite typically manifesting at an older age, can occasionally affect young women, whereas other forms of vasculitis such as giant cell arteritis are less likely to impact pregnancy.^24,25^ Due to the rarity of these diseases, data regarding pregnancy are limited.

### Fertility

Fertility is generally preserved in SV. However, it is important to consider that high-dose pulses or high cumulative oral dose of cyclophosphamide, which are sometimes used to treat SV, can lead to premature ovarian insufficiency, causing infertility or early menopause. Indeed, international recommendations and guidelines underline the importance of a preventive approach using gonadotropin-releasing hormone agonist.^6,26^ In women with SV who wish to undergo ARTs, several aspects must be carefully evaluated. In fact, not only must the disease be in clinical remission, as in any other autoimmune rheumatic disease, but also the individual thrombosis risk, which is not limited to aPL positivity, must be assessed.^6,24^ Prophylaxis with low-dose aspirin (LDA) with or without low molecular weight heparin during ARTs procedures should be carefully considered according to patient individual risk.^26^ Please refer to Leavitt et al^7^ for additional information on ART in this supplement.

### Preconception Counseling

Given that active disease could be associated with adverse pregnancy outcome (APO) in patients affected by SV, pregnancy planning is crucial.^24,27^ Similar to other rheumatic disease such as systemic lupus erythematosus (SLE), the aim should be to achieve and maintain low disease activity or remission in the 6 months before conception (Fig.).

Some medications used to treat SV are not compatible with pregnancy. Therefore, patients should be advised to avoid pregnancy and counseled on effective and safe contraception methods, considering both general and disease-related risk factors for thrombosis.^6,28,29^ Even in the absence of solid data focusing on the thrombotic risk of SV patients taking combined hormonal pill, we can expect an increased risk in these women because sometimes the thrombosis rate is increased and not limited to the acute phase of the disease. Therefore, estrogen containing pills should be avoided in these cases, and intrauterine devices or progestin-only pill should be preferred.^30^ Please refer to Lcuche et al30 for additional information on contraception in this supplement. It is important to remember that, if needed, emergency contraception is available and is not contraindicated in these patients.^24^

If the disease is well controlled and the patient desires a pregnancy, switching to pregnancy-compatible drugs should be considered during preconception counseling, when needed, to assess both the efficacy and tolerability of the new drug before pregnancy. Although data are available for some drugs, unfortunately for other more recently introduced drugs (eg, mepolizumab, avacopan), data are limited, if not absent, so they should be avoided.

Finally, pregnancy should be discouraged in cases of severe organ involvement, such as pulmonary hypertension, renal failure, heart failure, and recent stroke, given the high risk of maternal complications and mortality.^6^

### Pregnancy

Given the possibility of disease exacerbation during pregnancy, multidisciplinary management and close monitoring throughout pregnancy are essential. This includes also regular evaluation of blood and urine tests, as well as regular monitoring of arterial blood pressure. It is important to consider that, in patients with TAK and arterial stenosis, elevated blood pressure could be underestimated. In these cases, the assessment should be performed on a limb without stenosis or monitored in all limbs.^24,31^ Moreover, it needs be considered that some physiological changes occurring during pregnancy, such as increase intravascular volume or increase glomerular filtration, could have a negative impact on patients with a history of renal and/or cardiopulmonary involvement.^24,25^ Unfortunately, no modified-pregnancy indexes are available to assess disease activity during pregnancy in women with SV.^27^

A recent SLR including 8 studies for a total of 82 pregnancies in 64 patients with AAV reported disease flares in 25% of cases during pregnancy. In the majority of cases (56%), flares were mild to moderate (ear-nose-throat symptoms), whereas in 44% of cases, they were severe (including tracheal stenosis, subglottic stenosis, hemoptysis, progressive airway disease, renal disease), requiring surgery or an escalation of treatment.^32^ Notably, 5 flares (31%) occurred in the postpartum period, which should therefore be closely monitored in these patients.^32^

Data about disease activity during pregnancy in patients with BD are limited. An SLR by Ben-Chetrit in 2014 showed that the majority of patients either improved during pregnancy or did not experience changes in disease activity, whereas 27% had disease exacerbation.^33^ Interestingly, 1 of the included studies showed a higher rate of flares during pregnancy in patients with shorter disease duration.^33,34^ Oral and genital aphthosis and erythema nodosum were the manifestations that most frequently worsened during pregnancy, whereas thrombosis was rare.^33^ Overall, considering more recent studies, the frequency of BD flares during pregnancy seems to be low, with remission maintained in most cases.^33,35–37^ When flares did occur, they were mild, mainly represented by oral-genital ulcers or skin manifestations.^33,35–37^ Nevertheless, it should be noted that, in a few cases, severe flares such as neurological manifestations, ocular involvement, or transient ischemic attack occurred.^33,35–37^

In case of disease exacerbation during pregnancy, several therapeutic options can be considered, depending on the diagnosis and the severity and type of organ involvement. These options range from the use of high doses of steroids to the use of rituximab or cyclophosphamide in case of life-/organ-threatening conditions.^6,16,17^ In selected cases of severe glomerulonephritis in patients with AAV, plasma exchange could also be considered, although data on its use in this context are limited.^25^

It is therefore evident that the approach must be individualized. Among adjunctive treatments, thrombotic prophylaxis with LDA with or without low molecular weight heparin should be considered in every patient with SV, accounting for both general and disease-related risk factors. In this context, the use of LDA treatment is important also to prevent preeclampsia and other fetal complications.^24,25^

### Obstetric and Fetal Outcome

In general, pregnancy outcomes in patients with SV are favorable, and most women with vasculitis can experience a successful pregnancy.^24^ However, some complications such as preterm delivery, fetal loss, IUGR, and hypertensive disorders of pregnancy have been described and must be considered during preconception counseling and multidisciplinary follow-up.^24^

A recent SLR with meta-analysis, including 6 studies on pregnancy in women with AAV, reported preterm delivery in 18% of cases and a prevalence of IUGR of 20%.^38^ In a subsequent SLR, it was found that preeclampsia occurred in 16% of 64 pregnancies among patients with AAV, with 1 case progressing to HELLP (hemolysis, elevated liver enzymes, and low platelets) syndrome.^32^

Regarding TAK, an SLR with meta-analysis that included 27 studies with a large number of pregnancies, revealed a miscarriage rate of 16%.^39^ The overall prevalence of hypertension and preeclampsia was 37% and 14%, respectively.^39^ However, as discussed by the authors, it has to be considered that the heterogeneity of the results was significant, and high-quality observational studies involving patients with rare rheumatic diseases are needed.^39^ Finally, regarding the mode of delivery, the risk of aortic dissection or aortic regurgitation in patients with TAK may warrant the decision to proceed with a cesarean section.^40^

Among patients with BD, a high frequency of miscarriages and cesarean sections has been described, although some studies have not found differences between BD patients and controls.^33^ Moreover, fetal growth restriction, preeclampsia, preterm birth, hemorrhage, and gestational diabetes can occur.^35–37,41^

Given the frequency of preeclampsia in patients with SV, pregnancy prophylaxis with LDA should be considered, and patients should be regularly monitored, in particular in cases of previous renal involvement.^42^ In this context, differential diagnosis between glomerulonephritis and hypertensive disorders of pregnancy must be carefully evaluated by a multidisciplinary team.^24,25^

## SJÖGREN SYNDROME

SS occurs primarily in middle-aged women, of whom only 29% are younger than 45 years old at the time of diagnosis. Earlier onset in women of childbearing age is associated with more systemic manifestations and, consequently, more systemic immunosuppressive treatments. Clinicians must offer an accurate preconception counseling to women who desire to become mothers.

### Fertility

Data on fertility in patients with SS are controversial. Advanced age at diagnosis and possible treatment with cyclophosphamide may be possible causes of infertility in these patients. Pregnancy may also be delayed due to gynecological conditions such as menstrual irregularities, endometriosis, adenomyosis, and hormonal imbalance, which may occur more frequently in patients with connective tissue diseases.^43^ Indeed, an influence of SS on fertility and menstruations has been reported, mainly due to reduction in estrogen levels.^44^ A large Chinese cohort study evaluated fertility in 449 patients with primary SS (pSS), enrolled from 2015 to 2021: most patients experienced normal-age menarche, but early menopause was observed more frequently than in the general population.^45^ Moreover, the difficulties of sexual relationships in SS, especially in young women, are characterized by vaginal dryness and dyspareunia. These intimacy issues in young patients with SS could also be attributed to hormonal dysfunction, chronic fatigue, and depression.^46^

One additional point to note is that advanced maternal age could lead to more frequent use of ARTs, generally considered safe in patients with quiescent rheumatic diseases. ARTs do not present specific issues for women with SS, when the presence of aPL is excluded, and they can be managed according to the international guidelines and recommendations, as outlined elsewhere.

A recent a multicenter study evaluating 24 pregnancies in pSS patients who underwent ARTs showed that, overall, the outcome was favorable, with a high rate of live births, no complications related to the ART procedures, and no disease flares.^47^ Comparing these pregnancies with 70 naturally conceived pregnancies in pSS patients and 96 pregnancies in healthy individuals who underwent ARTs, the risk of fetal loss was similar across all 3 groups, whereas preterm delivery occurred more frequently in ARTs pregnancies in pSS.^47^ Birthweight was lower in offspring from ARTs pregnancies in pSS, but no differences were found in the rate of low-birth-weight neonates among the 3 groups.^47^

### Preconception Counseling

As general rule, disease should be well-controlled in the 6 months before conception, with the use of medications compatible with pregnancy (Fig.). Therefore, in cases of active disease or treatment with noncompatible drugs, pregnancy should be postponed. In these situations, contraception should be recommended. According to American College of Rheumatology guidelines,^6^ all contraceptive methods can be prescribed to women with SS if they test negative for aPL. The choice can be made based on the desired effective and the patient's preference.

### Pregnancy

Pregnancies in SS patients need to be regularly monitored by a multidisciplinary team (including rheumatologist, gynecologist, obstetric, and others) to promptly identify potential maternal or fetal complications.

An important concern during pregnancy in women with SS is maternal positivity for anti-Ro/SS-A and anti-La/SS-B antibodies, which can cross the placenta starting from the 11th week of gestation, potentially leading to neonatal lupus. The most common manifestations of LN are mild and include transient neonatal rash, transient hepatic abnormalities, or cytopenias.^6,48^ The most serious manifestation of neonatal lupus is cardiac involvement, characterized by the development of congenital heart block (CHB). However, not all offsprings of anti-Ro/SS-A– and/or anti-La/SS-B–positive women develop CHB. Other factors, such as antibodies titers, genetic predisposition, and ethnicity may play a role in the development of CHB.^49,50^ It is important to note that CHB resulting from maternal autoantibodies is rare, with reported cases in the literature ranging from 1%–2%, but the recurrence rate can be higher, up to 19%.^49,51,52^ Recently, an Italian prospective cohort study investigating pregnancy outcome in women with autoimmune disease showed that among the 866 pregnancies included, CHB was diagnosed in 2 cases out of 157 pregnancies in women with anti-Ro/SS-A antibodies: 1 in an asymptomatic carrier and 1 in a patient with SLE.^53^ No cases were observed among 40 patients with pSS. Even if the risk of first CHB is relatively low, women with anti-Ro/SS-A and/or anti-La/SS-B antibodies should be advised to undergo weekly or biweekly fetal echocardiography, between the 16th and the 26th week of gestation.^6,26,54^ Despite concerns about this intensive monitoring in women without a previous history of CHB, in our experience, this practice is safe, generally well-accepted by patients, and can lead to early detection of CHB.^55^ However, no universal evidence-based guidelines exist for CHB management, and therapeutic strategies are still controversial. Indeed, the efficacy of fluorinated steroids, the most frequently used treatment as highlighted in a recent electronic survey, is still debated.^6,56^ Most guidelines recommend using hydroxychloroquine in all pregnant women with anti-Ro/SS-A and/or anti-La/SS-B positivity. This is supported by observations that hydroxychloroquine may prevent complications related to maternal antibody positivity, reduce the recurrence risk of CHB, and lower the risk of flares during pregnancy.^6,26,57–59^

Literature discussing the effects of pregnancy on SS is limited. One report describes acute mesangio-proliferative glomerulonephritis presenting during pregnancy, leading to end-stage renal failure.^60^ Another case report described worsening tubulointerstitial nephritis over the course of 3 consecutive pregnancies.^61^ Recently, disease activity in 93 pregnancies in pSS patients was evaluated in a French multicenter prospective cohort study.^62^ Overall, disease flares were not frequent (13% of cases), and they were mild, involving symptoms such as articular (9%), cutaneous (5%), glandular (4%), hematologic (3%), and pulmonary (1%). These flares rarely required treatment changes.^62^ No baseline parameters were found to predict flare onset during pregnancy. Notably, a higher frequency of anti-La/SSB positivity was observed in pregnancies without flare.^62^

### Obstetric and Fetal Outcome

Most studies have reported effects of SS on pregnancy. Interestingly, a recent small population study identified low preconceptional C4 levels in SS as a possible marker for APO, as already observed in pregnancies in patients with SLE or antiphospholipid syndrome.^63–65^

Overall, studies have reported a higher frequency of APO in SS pregnancies, including abortion, preterm deliveries, small for gestational age (SGA) neonates, IUGR, and a higher frequency of cesarean sections. However, no specific correlations with clinical symptoms, laboratory indicators, organ involvement, or ongoing therapies have been found.^66,67^

It is important to note that many studies included women with SS associated with other connective tissue diseases and were mostly based on retrospective data from small cohorts. Data from larger cohorts on pSS seem to be reassuring. In the aforementioned French study, APO occurred in 6 out of 88 pregnancies included (7%): 2 IUGR, 1 intrauterine fetal death, 1 preeclampsia, 1 placental abruption, and 1 SGA neonate.^62^ A higher frequency of aPL positivity was observed in the group with APO. Interestingly, in the match-controlled analysis including 105 pSS pregnancies and 420 pregnancies in matched controls, the frequency of APO was similar between the 2 groups (9% and 7%, respectively).^62^ On the other hand, a meta-analysis including 1586 pregnancies in 544 pSS patients showed that women with pSS had a higher risk of overall fetal loss (OR, 1.77; 95% CI, 1.28–2.46) compared with the general population. However, it must be considered that the causes were not evaluated, and the majority of studies reporting this event were published in the 1990s.^68^ In the same study, other complications such as preterm births, abortion, and stillbirth were not significantly prevalent in women with pSS.^68^

A recent meta-analysis including 9 studies with a total number of 2472 pregnancies from 2341 SS patients showed a lower live birth rate, higher adverse maternal outcome (gestational hypertension, preeclampsia, cesarean section, and premature rupture of membrane), and adverse neonatal outcome (preterm delivery, SGA, IUGR, spontaneous abortion) in SS patients as compared with controls. No higher risk of stillbirth and neonatal death was found.^69^ Certainly, as highlighted by authors, several intrinsic limitations in the study can lead to these results, but other reasons related to SS can be posited: for example, endothelial dysfunction as well as the frequent occurrence of aPL positivity in SS patients may result in abnormal placentation and, consequently, in the onset of hypertensive disorders of pregnancy and/or miscarriages.^69,70^

Finally, a close monitoring is recommended in the postpartum period, as cases of disease flare may occur, especially in patients with cardiopulmonary involvement.^71^

## CONCLUSIONS

Although scarce or controversial, these data overall highlight the importance of a preconception counseling and multidisciplinary management during pregnancy in patients with SSc, SV, and SS, as in the other systemic autoimmune diseases. This approach is fundamental for personalizing individual management based on each patient's specific risk factors. Additionally, sharing decision with patients, their families, and colleagues from other specialties is recommended. These strategies could help minimize the risk of maternal and fetal complications during pregnancies, even in these complex and rare diseases.
